# Breast and prostate cancer: an analysis of common epidemiological features in mortality trends in Spain

**DOI:** 10.1186/1471-2407-14-874

**Published:** 2014-11-24

**Authors:** Gonzalo López-Abente, Sergio Mispireta, Marina Pollán

**Affiliations:** Environmental and Cancer Epidemiology Unit, National Centre for Epidemiology, Carlos III Institute of Health, Monforte de Lemos 5, 28029 Madrid, Spain; Consortium for Biomedical Research in Epidemiology and Public Health (CIBERESP), Madrid, Spain; Preventive Medicine Service, La Paz University Hospital, P° de la Castellana 261, 28046 Madrid, Spain

**Keywords:** Breast cancer, Prostate cancer, Epidemiology, Age-period-cohort, Spain

## Abstract

**Background:**

Breast cancer in women and prostate cancer are the first and second leading tumour respectively in terms of incidence world-wide. Our objective is to ascertain the similarities and differences between mortality trends in breast cancer among women and prostate cancer in Spain using age-period-cohort models, and analyse the correlation between incidence of breast and prostate cancer at cancer registries locally and world-wide.

**Methods:**

We analysed the independent effects of age, period of death and birth cohort on mortality rates for breast cancer in women and prostate cancer in Spain across the period 1952–2011. Segmented regression analyses were performed to detect and estimate changes in period and cohort curvatures. Correlation among age-adjusted incidence rates at 246 population cancer registries world-wide was analysed for the period 2003–2007.

**Results:**

The mortality trend displayed common characteristics in terms of the annual number of deaths due to these tumours, their adjusted mortality rates and the change points detected in the cohort and period effects. The trend in incidence was very different to that in mortality, due to early detection and progressive improvement in survival. Correlation between the incidence rates of both tumours recorded by registries around the world proved to be a generalised phenomenon.

**Conclusions:**

This study shows that breast cancer mortality in women and prostate cancer mortality and their trends in Spain display visible similarities in terms of the number of deaths due to these tumours, their adjusted mortality rates and the changes experienced by mortality over time. The effects of advances in the diagnosis of both tumours correspond to a decline in mortality which becomes evident after a lag of approximately eight years. Correlation between breast and prostate cancer incidence rates is very high in Spain and at registries on all continents.

## Background

Breast cancer is the leading tumour in terms of incidence among women world-wide [1]. It is estimated that there were 1,676,633 new cases in 2012, causing over half a million deaths. Despite the increase in the efficacy of diagnostic and therapeutic techniques, mortality has undergone relatively moderate changes and there are many aspects of the pathogenesis of breast cancer that are not well understood.

While prostate cancer is the second leading tumour in terms of world-wide incidence among men, with 1,111,689 estimated new cases in 2012, coming just behind lung cancer (1,241,601), it nevertheless ranks first in incidence in Europe with 417,124 new diagnoses in 2012. Prostate cancer incidence witnessed a steep rise in the 1990s in different countries, something that is attributed to the use of prostate-specific antigen (PSA) and thus viewed as an increase in detection [2, 3].

Observation of the coincidence between the biological, genetic and epidemiological aspects of breast and prostate cancer dates back to the 1950s. Already at that time, pioneering studies designed to ascertain the genetic bases of breast cancer (Macklin MT 1954) detected a higher frequency of prostate cancer among the relatives of women with breast cancer, which led them to propose that prostate cancer could be the male equivalent of at least some female mammary carcinomas.

In 1989, an extensive review was published on the epidemiological and aetiopathogenic similarities between both tumours, with documented explanations of this phenomenon [4]. One of most widely recognised characteristics is the role of hormonal regulation. Some types of breast and prostate cancer cells have receptors for similar steroid hormones and hormonal growth factors. The negative impact of high blood levels of endogenous sex steroids and the benefit of the low levels of these hormones in both tumours are known [5, 6], and it has been suggested that exposure to exogenous hormones (i.e., hormone therapy, contraceptives and environmental endocrine disruptors) may contribute to the onset and progression of both tumours.

This same review devoted a section to comparing the frequency of both tumours in 21 countries, showing the existence of a high correlation between the incidence rates of both tumours over a wide range of incidence [7]. This correlation supports the hypothesis of common causal pathways, probably including endogenous susceptibility and constitutional factors (hormonal, metabolic and genetic). Furthermore, the wide range of rates is an indication of the probable impact of various environmental risk factors.

With regard to genetic susceptibility, recent studies have confirmed the existence of common genetic variants associated with both tumours. Hence, the research groups that took part in the Collaborative Oncological Gene-environment Study (COGS) have shown that there are 18 loci in chromosomes associated with more than one of the hormone-dependent cancers (breast, ovarian and prostate). In addition, these studies, which included 160 research centres, established the contribution of low-penetrance polymorphic variants to individual susceptibility to developing cancer. The COGS almost doubles the number of identified common genetic variants that are significantly associated with susceptibility to breast, prostate and ovarian cancers [8, 9].

Accordingly, the aim of this study was: primarily, to ascertain the similarities and differences in mortality between breast cancer in women and prostate cancer in Spain using age-period-cohort models, and to study the trends in their respective rates; and, as a secondary objective, to analyse the correlation between incidence of breast and prostate cancer at cancer registries in Spain and around the world.

## Methods

### Mortality, population and incidence data

Mortality data for study purposes were obtained from the Spanish National Statistics Institute (*Instituto Nacional de Estadística*). During the calendar period considered (1952–2011), three different Revisions of the International Classification of Diseases (ICD) were used. Consequently, the cancer-related deaths studied corresponded to: ICD-6-7 code 170, ICD-8-9 code 174 and ICD-10 code C50 for breast cancer in women; and ICD-6-7 code 177 ICD 8–9 code 185 and ICD-10 code C61 for prostate cancer. These mortality data are publicly accessible. Spanish population data corresponding to censuses and municipal electoral rolls for the midyear of each quinquennium were also obtained from the National Statistics Institute. Mortality and population data were stratified by age group (from 0–4 to 85+ years), sex, calendar period (in twelve 5-year periods, i.e., 1952–1956, 1957–1961,…, 2007–2011), and cancer site. Age-adjusted mortality rates (per 100,000 population, standardised to the European Standard Population) for cancers of breast and prostate were calculated for each 5-year calendar period.

The time series of age-adjusted incidence rates in Spain for both tumours were obtained from references [10] and [11]. Note that these data cover the period 1981–2004 for breast cancer and cancer of prostate 1975–2004.

### Age-period-cohort (APC) models in mortality

Separate log-linear Poisson models were fitted to study the effect of age, period of death and birth cohort on mortality for each tumour site. Age-specific mortality rates per 100,000 population for the above twelve 5-year periods were used for the APC analysis. To address the "non-identifiability” problem (i.e., the three factors -age, period and cohort- are linearly dependent), we used curvature effects as proposed by Holford [12]. The following two estimable parameters not affected by the non-identifiability problem can be determined: (i) overall change over time (denominated net drift), which is the sum of the cohort and period slopes; and (ii) deviation of any period or cohort estimators from the general trend (denominated curvature). Net drift is of limited interest in the presence of change points. To display the cohort and period effects graphically, we used the respective curvatures. Ages <25 years for breast cancer and <40 years for prostate cancer, were excluded from this analysis due to the limited number of deaths in these age groups. The open-ended category of persons aged 85 years and over was also excluded. We checked for extra-Poisson dispersion [13], and effects were calculated using the negative binomial distribution.

### Curvature change points

The presence, both of change points in the age-adjusted mortality and incidence rates, and of curvatures of the cohort and period effects in mortality, was evaluated by fitting segmented models to the relationship between curvature effect and time. The models provided: 1) the estimate and 95% confidence interval for the location of the change point; and 2) the segments’ slope. Details of the algorithm used in the segmented regression have been published elsewhere [14], and the procedure was applied using the library “segmented” for the R programme [15]. It should be noted that, since the overall linear slopes were removed from the period and cohort curvatures, the specific slopes determined within each curvature segment only represent linear departures from the overall trend in mortality.

### Incidence rates from cancer registries

Data on the incidence of both tumours at the various registries around the world were drawn from Cancer Incidence in Five Continents (CIFC), Volume X [16]. The age-adjusted incidence rates for the period 2003–2007 were then computed (Standard European Population) for each registry and represented graphically and their breast-prostate cancer Pearson correlation coefficients and confidence intervals calculated.

## Results

Table 1 shows the number of deaths and age-adjusted mortality rates for both tumours by five-year period (1952–2011). The most noteworthy feature was the similarity between the tumours in terms of the magnitude of both indicators over the course of the twelve quinquennia.

**Table 1:** **Age-adjusted mortality rates per 100,000 person-years (European standard population) and number of deaths for breast cancer in women and prostate cancer per quinquennium, Spain 1952-2011**

	1952-56	1957-61	1962-66	1967-71	1972-76	1977-81	1982-86	1987-92	1993-96	1997-2001	2002-06	2007-11
Breast cancer												
Deaths	5053	6711	9323	11115	14158	17240	20966	26143	29117	28730	29100	30690
Rate	8.5	10.3	13.1	14.3	16.9	18.9	20.8	23.9	24.3	21.4	19.2	17.8
Prostate cancer												
Deaths	4039	6212	8878	10820	12683	15038	17508	20831	25319	27925	27847	28442
Rate	10.7	15.1	18.5	20.0	21.6	22.5	21.8	22.5	24.2	23.1	19.9	17.3

**Figure 1:**
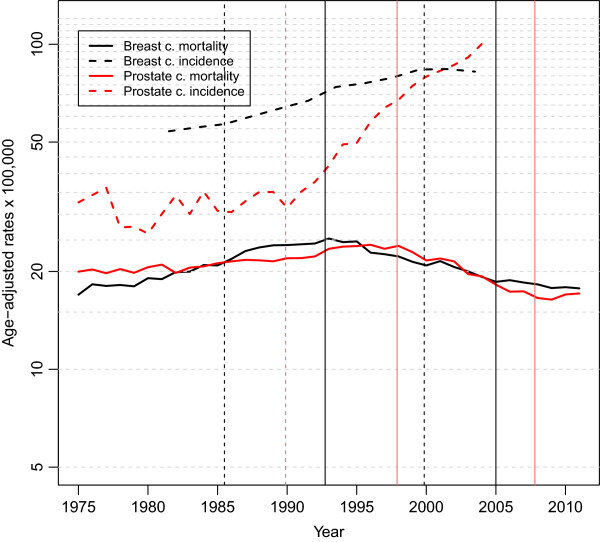
**Age adjusted rates of breast and prostate cancer incidence and mortality in Spain.** Years of change point are indicated with vertical lines, dashed for incidence and continuous for mortality.

Figure 1 plots the year-to-year trend in the adjusted mortality (1975–2011) and incidence rates, and their change points, for both tumours in Spain. It will be seen that, while the figure reflects the coincidence between the mortality rates, this was not so in the case of the incidence rates.

The change points detected in the incidence and mortality trends are denoted by vertical strokes. Two change points were detected in breast cancer incidence, in 1985 and again in 2000 (Table 2). It is of interest to see the sequence of changes in incidence and mortality: hence, the first change in the incidence trend in 1985 was followed by a change in the mortality trend in 1993, some eight years afterwards; similarly, the change point in incidence in 2000 was followed by a subsequent shift towards stabilisation of the mortality rates in 2005.

**Table 2:** **Points of change in age adjusted incidence and mortality rates on breast cancer in women and prostate cancer, Spain 1952–2011**

	AC% (95% CI)	Year (95% CI)	AC% (95% CI)	Year (95% CI)	AC% (95% CI)
Mortality					
Breast cancer	2.292 (2.089, 2.496)	1993 (1992–1993)	−2.381 (−2.738, −2.023)	2005 (2002–2008)	−1.118 (−1.933, −0.296)
Prostate cancer	0.902 (0.768, 1.037)	1998 (1997–1999)	−3.655 (−4.134, −3.174)	2008 (2007 – 2009)	2.204 (−1.016, 5.529)
Incidence					
Breast cancer	1.379 (−1.446, 4.286)	1985 (1980 – 1991)	2.831 (2.514, 3.148)	2000 (1998 – 2002)	−0.898 (−3.660, 1.944)
Prostate cancer	0.549 (−0.470, 1.578)	1990 (1988 – 1991)	8.593 (7.493, 9.705)		

AC%: annual percentage change.

The prostate cancer incidence trend displays a single change point in 1990. Incidence practically went from stability (0.5% per annum) to a sharp increase, with the slope increasing 16-fold (8.6% per annum) (Table 2). This change in incidence was followed by a change in the trend in mortality rates in 1998 (8 years later, the same lag as in breast cancer). In 2008, there was another upturn (not statistically significant) in the prostate cancer mortality trend. There is no way of knowing whether this upward shift in the mortality trend was preceded by some change in incidence, due to the break in the series in 2004.

Shown in Table 3 is the deviance table for the different log-linear models fitted for the two tumours. The model that displayed the best fit was that which contained the three components (age + period + cohort), with the period component being the one which most contributed to the improvement of the models in statistical terms, particularly in the case of breast cancer.

**Table 3:** **Goodness of fit for age-period-cohort models to breast and prostate cancer mortality, Spain 1952-2011**

	Breast cancer women		Prostate cancer	
Model	D.f	Deviance	D.f	Deviance
age	132	8568.7	99	1879.9
age + drift	131	4882.6	98	1876.5
age + per	121	1019.7	88	331.6
age + coh	110	2427.0	80	652.9
age + per + coh	100	172.7	70	115.5

D.f. Degrees of freedom.

**Figure 2:**
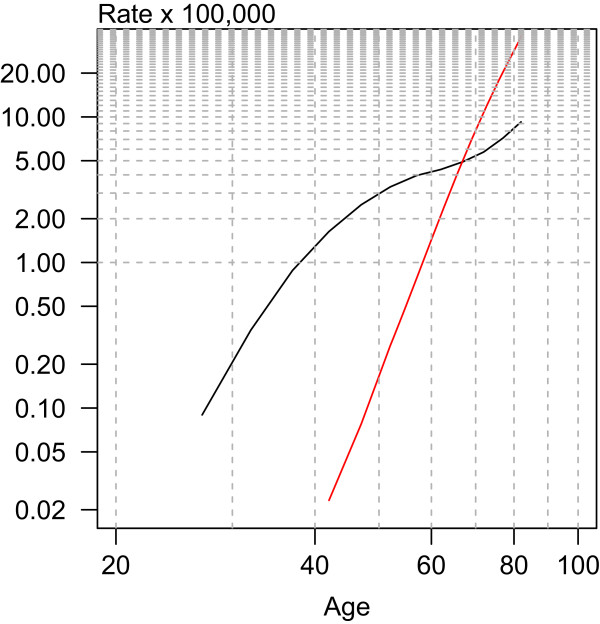
**Age effect for breast (black) and prostate (red) cancer mortality in Spain.**

Figure 2 depicts the age effect, which behaved very differently in the two tumours. Breast cancer registered rates higher than those of prostate cancer until age sixty years, with an inflection point in mortality around the age of menopause (Clemmensen’s hook). The rate at which mortality increased with age declined after menopause. In prostate cancer, however, the increase in mortality with age was exponential.

**Figure 3:**
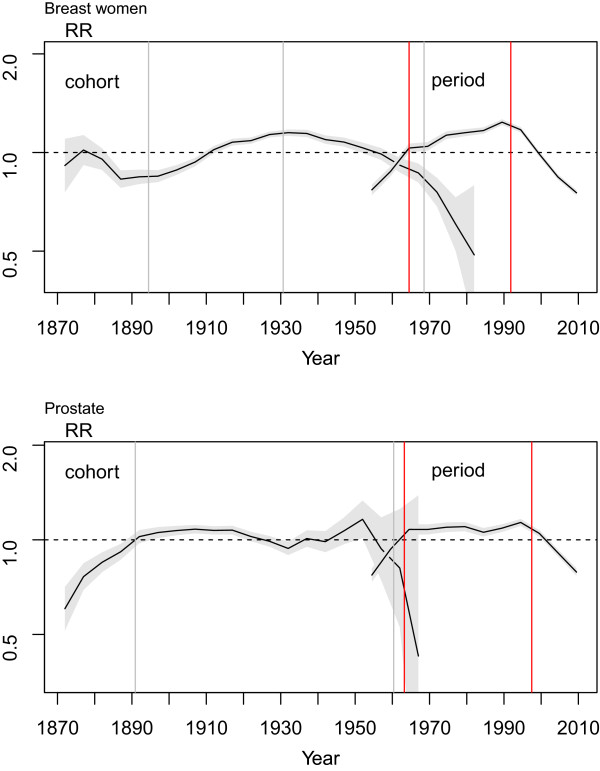
**Cohort and period effect curvatures and 95% confidence interval (shadow) for breast and prostate cancer mortality in Spain.** Years of change point are indicated with vertical lines, grey for cohort effect and red for period effect.

Figure 3 plots the curvatures of the cohort and period effects. In breast cancer, the cohort effect displayed three change points, i.e., in 1894, 1931 and 1969; and, while the “shape” of the cohort effect was different in prostate cancer, there was a certain coincidence in change-point years.

The curvature of the period effect was more similar between the two tumours, with a first change point which can be interpreted as consolidation of the registration of mortality in both breast and prostate cancer, and a second change point which coincides with that already described for mortality and confirms the decline in mortality due to these tumours. The specific results of this analysis are shown in detail in Table 4.

**Table 4:** **Cohort and period effect curvature points of change on breast cancer in women and prostate cancer mortality, Spain 1952–2011**

	Changes in cohort effect curvature						
	Slope* (95% CI)	Birth year (95% CI)	Slope (95% CI)	Birth year (95% CI)	Slope (95% CI)	Birth year (95% CI)	Slope (95% CI)
Breast cancer	−0.007 (−0.002, −0.012)	1894 (1889–1900)	0.010 (0.007, 0.013)	1931 (1926–1935)	−0.008 (−0.011, −0.006)	1969 (1965–1972)	−0.044 (−0.056,-0.032)
Prostate cancer	0.027 (0.016, 0.038)	1891 (1886–1899)	−0.001 (−0.002, 0.001)			1960 (1959–1962)	−0.128 (−0.163, −0.091)
	**Changes in period effect curvature**						
		**Year of death (95% CI)**		**Year of death (95% CI)**			
Breast cancer	0.029 (0.015, 0.043)	1965 (1960–1969)	0.007 (0.005, 0.01)	1992 (1990–1993)	−0.030 (−0.034, −0.025)		
Prostate cancer	0.038 (0.024, 0.051)	1963 (1961–1965)	0.001 (−0.001, 0.003)	1998 (1996–1999)	−0.028 (−0.035, −0.022)		

*Slopes of each ‘segment’ in the curvature.

**Figure 4:**
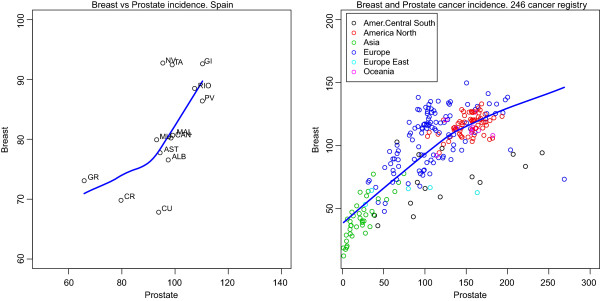
**Correlation between breast cancer and prostate cancer incidence in Spanish cancer registries (left) and in 246 registries from all over the world (rigth) (2003–2007).** The blue line is a locally weighted scatterplot smoothing (loess). (Source: [16]). NA: Navarra, GI: Girona, RIO: La Rioja, PV: País Vasco, MAL: Mallorca, MU: Murcia, CAN: Cantabria, AST: Asturias, ALB; Albacete, GR: Granada, CR: Ciudad Real, CU: Cuenca.

Figure 4 shows the correlation between the incidence of breast and prostate cancer. The correlation between the incidence of both tumours at cancer registries in Spain and other countries was analysed using data drawn from the CIFC, Volume X. Correlation coefficient was 0.65 (95% CI 0.15, 0.88) at 13 Spanish registries and 0.76 (95% CI 0.71, 0.81) at 246 registries world-wide. While rates in Spain ranged from 67.8-92.8 cases per 100,000 for breast cancer and from 65.8-110.3 per 100,000 for prostate cancer, those at registries around the world ranged from 12.5-159.8 per 100,000 for breast cancer (30.57-159.8 excluding China and Thailand due to their extremely low rates) and from 1.3-268.8 per 100,000 for prostate cancer (17.1-268.8 excluding China and Thailand). The highest breast cancer rates were registered in Europe by Italy, France, Switzerland, The Netherlands, Germany and Belgium, and in the USA and Canada. The lowest rates were found at registries corresponding to Asian countries.

## Discussion

This study explores similarities and differences in mortality trends for breast cancer in women and prostate cancer in Spain. The mortality trend displays common characteristics in terms of the annual number of deaths due to these tumours, their adjusted mortality rates and the changes seen in mortality over time. The incidence trend is very different to that of mortality. The peculiarities of the changes in both indicators are discussed below.

The magnitude of the **incidence** rates and their trend are different in the two tumours in Spain, as can be seen in Figure 1. Even so, both the international data and the different registries around Spain show a high correlation in the incidence of these tumours. Furthermore, both tumours display change points followed by increases in incidence probably associated in part with early detection [10, 17]. Early detection can lead to overdiagnosis and overtreatment phenomena with consequences in incidence and mortality [18]. For the moment, we don’t know the magnitude of the problem in Spain, although we could asume that the magnitude of overdiagnosis in our country could be similar to those recently reported for neighbouring countries, between 2.8% in the Netherlands and 4.6% in Italy, two countries with biennial screening programmes of breast cancer [19].

Any advance in the diagnosis of these tumours generally implies better management and prognosis, which in turn translates as a decrease in mortality. The sequence of change points resulting from the increase in detection and subsequent decrease in mortality occurs at a lag of 8 years in both tumours. This similarity in lags might indicate the period needed for the generalisation of early detection methods to be translated into an increase in survival and, by extension, into a decrease in mortality, though the latter calls for more in-depth analysis of the factors that might be associated with these two indicators in the tumours studied and for comparison with detection strategies applied to other tumours.

With respect to the trend in breast cancer incidence rates, the first change point could in part be explained by the progressive increase in detection caused by the implementation of screening programmes, while the second point, at which the trend stabilises, has been interpreted as the saturation of the respective screening programmes [11]. The first breast cancer screening programme was initiated in Navarre in 1990. This was followed in 1992 by Castile-La Mancha, Catalonia, Galicia and the Valencian Region, and in subsequent years by the remaining Autonomous Regions (*Comunidades Autónomas*). The first point denoting a change in the increase in incidence in 1985 precedes the introduction of screening programmes, which suggests that early “opportunistic” detection was already showing its effect.

Insofar as the trend in prostate cancer incidence is concerned, in Spain there are no specific recommendations regarding early detection of this tumour, though different studies [20, 21] show that opportunistic use of PSA as a screening test intensified at the end of the 1990s and its use has since become very widespread. The sharp change observed in the incidence of this tumour could be connected, as in the case of breast cancer, with the early implementation of such active case searching (opportunistic practices) and a higher degree of awareness among the population and professionals alike. Better access to health services and the introduction into routine clinical practice of therapeutic modalities such as transuretral resection and diagnostic procedures such as echo-guided biopsy, transrectal ultrasonography in addition to PSA testing, can be assumed to have made a greater contribution to this increase as a result of an enhanced capability to detect incidental cancers that would otherwise be latent [10].

The great difference between the incidence of these cancers and mortality reflects enhanced patient survival. This was documented on the basis of the most recent Eurocare-5 results (2000–2007) [22], which indicate a relative survival at 5 years of 84.48% (95%CI: 83.62-85.35) and 85.18% (95%CI: 84.52-85.84) for prostate and breast cancer respectively, percentages which in both cases are higher than those observed in earlier periods.

The results show that there are similarities in breast cancer **mortality** in women and prostate cancer mortality, and their trends in Spain in terms of the annual number of deaths, adjusted mortality rates and changes plotted by this indicator across the study period.

From the comparative analysis of the trend in the adjusted mortality rates, it is clear that the most important change which took place was the decrease that occurred after 1993 in breast cancer and 1998 in prostate cancer. As mentioned above, it is the improvement in prognosis stemming from advances in detection, combined with a better therapeutic strategy, that might largely underlie the decline in mortality of both tumours.

Using age-period-cohort models to analyse the mortality trend enables the similarities of the three components to be assessed. In the first place, the effect of age is very different. Prostate cancer mortality affects more advanced age groups than does breast cancer mortality. In addition, hormonal changes specific to menopause determine one aspect (“shape”) of the very characteristic age effect in breast cancer.

Analysis of the **period effect** shows that the change points occur in similar years in both tumours. The period effect, moreover, is comparatively more important, as is shown by its greater influence in improving the goodness-of-fit of the models (Table 3), principally in the case of breast cancer. A first change point occurred in the period 1963–1965 in both tumours, which might correspond to the consolidation of mortality statistics in Spain. A second change point, with a difference of 5 years between the two tumours, coincides with the decrease in mortality in the adjusted rates. Changes identified through the period effect tend to be phenomena that affect a wide range of age groups, as happens with changes in the registration, diagnostic criteria and treatment of these tumours.

The decline in mortality in both tumours is partly due to benefits deriving from early detection. In the case of breast cancer, however, the benefits of new treatments also play an important role [23, 24]. Early diagnosis is widely accepted as a pre-requisite for a successful treatment. The striking differences in survival according to TNM stage support this statement. In the case of breast cancer, for example, a recent review of survival rates by stage at diagnosis carried out all around the country [25] showed that while 5-year survival for patients diagnosed in stage I was 96.5%, this percentage dropped to 29.2% for those in stage IV. Regardless of the relative weight of each of these components, the most likely explanation is that both early detection and therapeutic improvements jointly account for the second change in the curvature of the period effect.

The **cohort effects** of both tumours display some differences for which we have no explanation. Curvature in breast cancer registered a peak in the generations of women born in the period 1930–1940. In prostate cancer, the curvature of the cohort effect showed a lower indicator in the generations born in the years that coincided with the Spanish Civil War, though the change-point analysis indicated no variations detectable from a statistical standpoint. On examining the specific rates (age-specific), it would appear that the “valley” in the cohort effect might be caused by lower mortality in the age groups from 40 to 50 years and in the years of death from 1970 to 1980, which would make it difficult to distinguish whether this is an effect associated with year of death or a cohort effect. This difference in the cohort effect is maintained when an analysis is performed, including the same age groups (50 years and over) in both tumours (results not shown).

The similarities in the frequency of both tumours in 21 countries and the strong correlation in their incidence rates over a wide range of incidence is a well-known fact [7]. We have updated the analysis of the correlation of the incidence of both tumours at registries in Spain and abroad using data drawn from the CIFC, Volume X [16]. The highest incidence occurs in some European countries, together with USA and Canada, while the lowest is observed in Asia. The latter finding is especially suggestive since, from a purely theoretical stance, pinpointing the environmental factors that induce this difference would afford an important opportunity for primary prevention. We are unaware to what extent the correlation between the rates of the two tumours might be due to environmental factors that could be assumed to act via common pathways of a hormonal nature in both tumours, to shared genetic susceptibility or, more probably, to a combination of both.

A family history of prostate cancer or breast cancer significantly increases prostate cancer risk and these associations are evident in a population with widespread PSA screening [26]. The newly susceptibility loci identified by the COGS account for an increasing proportion of the familial risk of such cancers [27]. Taking these new loci into account, the proportion of familial risk explained by common genetic loci is now estimated at 28% for breast cancer [8], 4% for ovarian cancer [28] and 30% for prostate cancer [9].

Bearing this information in mind, genetic susceptibility would only explain part of the similarities in the frequency of the two tumours. In contrast, high-income countries as well as urbanised and industrialised areas of middle- and low-income regions and countries have higher rates of colorectal cancer and hormone-related cancers (of the breast, ovary, endometrium and prostate), though this similarity is not seen in the case of Japan which, being a highly developed country, has very low breast cancer rates. The change in reproductive patterns characteristic of the most developed societies accounts for the increase in certain female hormonal tumours, such as those of the breast and endometrium, whereas the use of exogenous hormones is also associated with an increase in these tumours and a lower risk of ovarian cancer [29].

The compilation of scientific data on the role of diet and physical activity put together by the World Cancer Research Fund in 2007 [30] makes it possible to review the conclusions of the assessment of knowledge of risk and protective factors in breast and prostate cancer. Obesity and the distribution of body fat are risk factors for postmenopausal breast cancer and for the most aggressive tumours of prostate, which are precisely those that display the worst survival [30, 31]. Overweight and obesity are an increasing problem in our country. According to data from the Spanish National Health survey, while 8% or women and 7% of men older than 17 were obese in 1987 these percentages have doubled by 2006 (15% in women and 16% in men). The problem is more marked in middle and older age. In 2006, 21% of men aged 45 or older were obese, while 19% of women in the age-range of 45–64 and 26% of those aged 65 and more were obese. These percentages are based on self-reported weight and height, so the real figures can be even worse.

Physical activity probably protects against post-menopausal breast cancer but the evidence is limited for pre-menopausal breast cancer, and the information is very limited for prostate cancer, though such activity is believed to protect against the most aggressive forms of this tumour [32].

At the same time, on examining dietary and cancer patterns around the world and among migrants, it has increasingly come to be thought that energy-dense foods, red meat and processed meat are involved in the etiology of some cancers, notably those of the colon and rectum and breast [33, 34].

Despite the many epidemiological studies that have addressed the role of certain foods and nutrients (apart from the harmful effect of alcohol for breast cancer) in both pre- and post-menopausal women [26], the results are extremely heterogeneous and there is no conclusive evidence. In this respect, a recent study in our country shows an association between a Western dietary pattern, characterized by high consumption of these type of foods, and breast cancer [35].

The link between diet and these tumours would presumably be mediated by the serum levels of sex hormones, since the levels of circulating oestrogens are known to change due to modifications in body mass index and other dietary factors. On the other hand, serum levels of circulating oestrogens are lower in Asian than in North-American or European populations [36]. Furthermore, the role of androgens in prostate cancer is widely acknowledged, and there are studies which indicate that oestrogens, alone or in synergy with androgens, may have a relevant role in the aetiology of prostatic hyperplasia and prostate cancer.

## Conclusions

This study shows that breast cancer mortality in women and prostate cancer mortality and their trends in Spain display visible similarities in terms of the number of deaths due to these tumours, their adjusted mortality rates and the changes experienced by mortality over time. Mortality age-effects also shows differences attributable to the respective hormonal changes that take place in men and women. The effects deriving from advances in the diagnosis of both tumours correspond to a decline in mortality detected at a lag of approximately eight years. The correlation between breast and prostate cancer incidence rates is very high both in Spain and at registries on all five continents.

## References

[1] Ferlay J, Shin H, Bray F, Forman D, Mathers C, Parkin D (2010). GLOBOCAN 2008, Cancer Incidence and Mortality Worldwide: IARC CancerBase No. 10 [Internet].

[2] Mandal R, St-Hilaire S, Kie JG, Derryberry D (2009). Spatial trends of breast and prostate cancers in the United States between 2000 and 2005. Int J Health Geogr.

[3] Mistry M, Parkin DM, Ahmad AS, Sasieni P (2011). Cancer incidence in the United Kingdom: projections to the year 2030. Br J Cancer.

[4] López-Otín C, Diamandis EP (1998). Breast and prostate cancer: an analysis of common epidemiological, genetic, and biochemical features. Endocr Rev.

[5] Cuzick J (2008). Hormone replacement therapy and the risk of breast cancer. Eur J Cancer.

[6] Prins GS (2008). Endocrine disruptors and prostate cancer risk. Endocr Relat Cancer.

[7] Prentice RL, Sheppard L (1990). Dietary fat and cancer: consistency of the epidemiologic data, and disease prevention that may follow from a practical reduction in fat consumption. Cancer Causes Control.

[8] Michailidou K, Hall P, Gonzalez-Neira A, Ghoussaini M, Dennis J, Milne RL, Schmidt MK, Chang-Claude J, Bojesen SE, Bolla MK, Wang Q, Dicks E, Lee A, Turnbull C, Rahman N, Fletcher O, Peto J, Gibson L, Dos Santos Silva I, Nevanlinna H, Muranen TA, Aittomäki K, Blomqvist C, Czene K, Irwanto A, Liu J, Waisfisz Q, Meijers-Heijboer H, Adank M, Breast and Ovarian Cancer Susceptibility Collaboration (2013). Large-scale genotyping identifies 41 new loci associated with breast cancer risk. Nat Genet.

[9] Eeles RA, Olama AAA, Benlloch S, Saunders EJ, Leongamornlert DA, Tymrakiewicz M, Ghoussaini M, Luccarini C, Dennis J, Jugurnauth-Little S, Dadaev T, Neal DE, Hamdy FC, Donovan JL, Muir K, Giles GG, Severi G, Wiklund F, Gronberg H, Haiman CA, Schumacher F, Henderson BE, Le Marchand L, Lindstrom S, Kraft P, Hunter DJ, Gapstur S, Chanock SJ, Berndt SI, Albanes D (2013). Identification of 23 new prostate cancer susceptibility loci using the iCOGS custom genotyping array. Nat Genet.

[10] Larrañaga N, Galceran J, Ardanaz E, Franch P, Navarro C, Sánchez MJ, Pastor-Barriuso R, Prostate Cancer Working Group (2010). Prostate cancer incidence trends in Spain before and during the prostate-specific antigen era: impact on mortality. Ann Oncol.

[11] Pollán M, Pastor-Barriuso R, Ardanaz E, Argüelles M, Martos C, Galcerán J, Sánchez-Pérez M-J, Chirlaque M-D, Larrañaga N, Martínez-Cobo R, Tobalina M-C, Vidal E, Marcos-Gragera R, Mateos A, Garau I, Rojas-Martín M-D, Jiménez R, Torrella-Ramos A, Perucha J, Pérez-de-Rada M-E, González S, Rabanaque M-J, Borràs J, Navarro C, Hernández E, Izquierdo A, López-Abente G, Martínez C (2009). Recent changes in breast cancer incidence in Spain, 1980–2004. J Natl Cancer Inst.

[12] Holford TR (1991). Understanding the effects of age, period, and cohort on incidence and mortality rates. Annu Rev Public Health.

[13] Dean C (1992). Testing for overdispersion in Poisson and binomial regression models. J Am Stat Assoc.

[14] Muggeo VMR (2003). Estimating regression models with unknown break-points. Stat Med.

[15] R Development Core Team (2005). R: A Language and Environment for Statistical Computing.

[16] Forman D, Bray F, Brewster D, Gombe Mbalawa C, Kohler B, Piñeros M, Steliarova-Foucher M, Swaminathan R, Ferlay J (2013). Cancer Incidence in Five Continents Vol. X, Volume 164.

[17] Ascunce N, Salas D, Zubizarreta R, Almazán R, Ibáñez J, Ederra M, Network of Spanish Cancer Screening Programmes (Red de Programas Espanoles de Cribado de Cancer) (2010). Cancer screening in Spain. Ann Oncol.

[18] Martinez-Alonso M, Vilaprinyo E, Marcos-Gragera R, Rue M (2010). Breast cancer incidence and overdiagnosis in Catalonia (Spain). Breast Cancer Res BCR.

[19] Puliti D, Duffy SW, Miccinesi G, de Koning H, Lynge E, Zappa M, Paci E, EUROSCREEN Working Group (2012). Overdiagnosis in mammographic screening for breast cancer in Europe: a literature review. J Med Screen.

[20] Páez A, Luján M, Llanes L, Romero I, de la Cal MA, Miravalles E, Berenguer A (2002). PSA-use in a Spanish industrial area. Eur Urol.

[21] Cepeda Piorno J, Rivas del Fresno M, Fuente Martín E, González García E, Muruamendiaraz Fernández V, Fernández Rodríguez E (2005). [Advantages and risks of the use of prostate-specific antigen (PSA) in the health-care area No. 4 of Gijon (Asturias)]. Arch Esp Urol.

[22] De Angelis R, Sant M, Coleman MP, Francisci S, Baili P, Pierannunzio D, Trama A, Visser O, Brenner H, Ardanaz E, Bielska-Lasota M, Engholm G, Nennecke A, Siesling S, Berrino F, Capocaccia R (2014). EUROCARE-5 Working Group: Cancer survival in Europe 1999–2007 by country and age: results of EUROCARE–5-a population-based study. Lancet Oncol.

[23] Kalager M, Adami H-O, Bretthauer M (2014). Too much mammography. BMJ.

[24] Autier P, Boniol M, Gavin A, Vatten LJ (2011). Breast cancer mortality in neighbouring European countries with different levels of screening but similar access to treatment: trend analysis of WHO mortality database. BMJ.

[25] Martín M, Pollán M, Jara C, López-Tarruella S, Carrasco E (2014). Proyecto EL Álamo III. Encuesta de evolución de pacientes con cáncer de mama en hospitales del grupo GEICAM 1998–2001.

[26] Chen Y-C, Page JH, Chen R, Giovannucci E (2008). Family history of prostate and breast cancer and the risk of prostate cancer in the PSA era. Prostate.

[27] Sakoda LC, Jorgenson E, Witte JS (2013). Turning of COGS moves forward findings for hormonally mediated cancers. Nat Genet.

[28] Pharoah PDP, Tsai Y-Y, Ramus SJ, Phelan CM, Goode EL, Lawrenson K, Buckley M, Fridley BL, Tyrer JP, Shen H, Weber R, Karevan R, Larson MC, Song H, Tessier DC, Bacot F, Vincent D, Cunningham JM, Dennis J, Dicks E, Aben KK, Anton-Culver H, Antonenkova N, Armasu SM, Baglietto L, Bandera EV, Beckmann MW, Birrer MJ, Australian Cancer Study, Australian Ovarian Cancer Study Group (2013). GWAS meta-analysis and replication identifies three new susceptibility loci for ovarian cancer. Nat Genet.

[29] Pike MC, Pearce CL, Wu AH (2004). Prevention of cancers of the breast, endometrium and ovary. Oncogene.

[30] World Cancer Research Fund/American Institute for Cancer Research (2007). Food, Nutrition, Physical Activity, and the Prevention of Cancer: A Global Perspective.

[31] Hsing AW, Sakoda LC, Chua S (2007). Obesity, metabolic syndrome, and prostate cancer. Am J Clin Nutr.

[32] Patel AV, Rodriguez C, Jacobs EJ, Solomon L, Thun MJ, Calle EE (2005). Recreational physical activity and risk of prostate cancer in a large cohort of U.S. men. Cancer Epidemiol Biomark Prev.

[33] World Cancer Research Fund / American Institute for Cancer Research (2011). Colorectal Cancer 2011 Report. Food, Nutrition, Physical Activity, and the Prevention of Colorectal Cancer.

[34] Alexander DD, Morimoto LM, Mink PJ, Cushing CA (2010). A review and meta-analysis of red and processed meat consumption and breast cancer. Nutr Res Rev.

[35] Castelló A, Pollán M, Buijsse B, Ruiz A, Casas AM, Baena-Cañada JM, Lope V, Antolín S, Ramos M, Muñoz M, Lluch A, de Juan-Ferré A, Jara C, Jimeno MA, Rosado P, Díaz E, Guillem V, Carrasco E, Pérez-Gómez B, Vioque J, Boeing H, Martín M (2014). Spanish Mediterranean diet and other dietary patterns and breast cancer risk: case–control EpiGEICAM study. Br J Cancer.

[36] Key TJ, Chen J, Wang DY, Pike MC, Boreham J (1990). Sex hormones in women in rural China and in Britain. Br J Cancer.

[CR37] The pre-publication history for this paper can be accessed here:http://www.biomedcentral.com/1471-2407/14/874/prepub

